# Isolation, Identification and Characteristics of an Endophytic Quinclorac Degrading Bacterium *Bacillus megaterium* Q3

**DOI:** 10.1371/journal.pone.0108012

**Published:** 2014-09-22

**Authors:** Min Liu, Kun Luo, Yunsheng Wang, Aiping Zeng, Xiaomao Zhou, Feng Luo, Lianyang Bai

**Affiliations:** 1 Institute of Pesticide Science, Hunan Agricultural University, Changsha, China; 2 College of Plant Protection, Hunan Agricultural University, Changsha, China; 3 School of Computing, Clemson University, Clemson, South Carolina, United States of America; 4 Hunan academy of agricultural sciences, Changsha, China; Institute of Vegetables and Flowers, Chinese Academy of Agricultural Science, China

## Abstract

In this study, we isolated an endophytic quinclorac-degrading bacterium strain Q3 from the root of tobacco grown in quinclorac contaminated soil. Based on morphological characteristics, Biolog identification, and 16S rDNA sequence analysis, we identified strain Q3 as *Bacillus megaterium*. We investigated the effects of temperature, pH, inoculation size, and initial quinclorac concentration on growth and degrading efficiency of Q3. Under the optimal degrading condition, Q3 could degrade 93% of quinclorac from the initial concentration of 20 mg/L in seven days. We analyzed the degradation products of quinclorac using liquid chromatography–tandem mass spectrometry (LC-MS/MS). The major degradation products by Q3 were different from those of previously identified quinclorac degrading strains, which suggests that Q3 may employ new pathways for quinclorac degradation. Our indoor pot experiments demonstrated that Q3 can effectively alleviate the quinclorac phytotoxicity in tobacco. As the first endophytic microbial that is capable of degrading quinclorac, Q3 can be a good bioremediation bacterium for quinclorac phytotoxicity.

## Introduction

Quinclorac (3,7-dichloro-8-quinoline-carboxylic) is a highly selective auxin herbicide developed by the BASF Corporation. It is widely used for controlling barnyard grasses and certain dicot grasses in rice fields [1]. Quinclorac can be used as both a pre-emergence and post-emergence herbicide. Quinclorac stimulates the induction of 1-aminocyclopropane-1-carboxylic acid (ACC) synthase, a key enzyme in ethylene formation, which leads to the accumulation of cyanide in the tissues of plants and ultimately—to their death [2], [3].

Meanwhile, quinclorac is highly stable, and degradation of quinclorac in nature is very slow [4]. The quinclorac residues are phytotoxic to many crops and vegetables, which has become one of the major problems in their rotation with rice [5]. Especially in a rice-tobacco rotation field, residues of quinclorac can cause serious phytotoxicity to sensitive tobacco, which has a strong effect on the yield and quality of tobacco. The quinclorac residues may also affect microbial activity and bring potential hazards to the environment through soil contamination [6]. Currently, two major methods are used to reduce quinclorac contamination. They are photodegradation and microbial degradation [7], [8]. Photodegradation is only useful when quinclorac exists in the soil's surface. On the other hand, microbial degradation is not limited by this constraint. Bioremediation using microbial degradation is an effective way to control pesticide residue [9]. Several quinclorac degrading strains were isolated from quinclorac-contaminated soil. Lü et al. isolated a WZ1 strain from pesticide manufactory soil, which was capable of degrading quinclorac [10]. WZ1 was identified as *Burkholderia cepacia* [10]. At optimal conditions, WZ1 degraded 90% of quinclorac from an initial concentration of 1000 mg/L in 11 days [10], [11], [12]. In 2012, Xu et al. also isolated a *Bordetella sp*. strain HN36 [13] from a pesticide manufactory soil. HN36 strain degraded 96% of quinclorac from an initial concentration of 400 mg/L in 48 h [13]. In 2013, Dong et al. isolated a quinclorac degrading J3 bacterium strain from soil with long-term quinclorac usage. The J3 was identified as *Alcaligenes sp* [14]. The J3 strain degraded 70% of quinclorac from initial concentration of 100 mg/L in 7 days at optimal conditions [14]. Fan et al. isolated a *Pantoea* sp. QC06 strain from soil with long-term quinclorac usage in 2013. The strain QC06 degraded 95.31% of quinclorac from initial concentration of 50 mg/L in 7 days at optimal degrading conditions [15].

Currently, no study has reported on the capability of the endophytic microbial bacterium to degrade quinclorac. In this paper, we isolated the Q3, an endophytic quinclorac-degrading bacterium, from the root of tobacco grown in a quinclorac-contaminated field. Compared with microbial strains isolated from highly concentrated quinclorac soil from pesticide manufactory, endophytic microbial strains are capable of degrading quinclorac in lower concentration, such as the concentration of quinclorac commonly found in a rice field [16]. We investigated its morphological, physiological and biochemical characteristics. We sequenced the 16S rDNA of Q3 and identified it as *Bacillus megaterium*. We explored the degradation conditions of Q3 to find its optimal degradation condition. We analyzed the microbial degradation products of quinclorac using HPLC-MS/MS. The degradation products showed that Q3 may employ degradation pathways that differ from those of previously identified quinclorac degrading microbial. We also conducted indoor pot cultivation experiments to demonstrate the bioremediation function of Q3 on tobacco quinclorac phytotoxicity.

## Materials and Methods

### Materials

Quinclorac standard (98.1%) was purchased from Dr. Ehrenstorfer GmbH (Germany. HPLC grade methanol was purchased from Tedia Company (USA). Other analytical reagents were bought from the China National Pharmaceutical Group Corporation, SINOPHARM.

The root of widely planted tobacco K326 was collected from the private land of Mr. Huibo Zhou in Shiyan village of Lougu Town, Liuyang City of Hunan Province. Mr. Zhou should be contacted for future permissions. The collection of root did not involve endangered or protected species and was safe to the environment. No specific permission is needed. The GPS coordinates of the field are E113.50′99.23″ and N28.36′ 34.24″.

The composition of mineral salt medium (MSM) was as follows (g.L^−1^): (NH_4_)_2_SO_4_ 1.0, KH_2_PO_4_ 2.0, MgSO_4_.7H_2_O 0.5, NaCl 0.1, and CaCO_3_ 0.5. For quinclorac degrading, 0.5 g sucrose and 0.5 g yeast powder were added to the mineral salt medium as an additional carbon source. The composition of Luria–Bertani's (LB) medium for purification and enlarged cultivation was as follows (g.L^−1^): yeast powder 5, tryptone 10, NaCl 10. In solid LB medium, 15 g.L^−1^ agar was added.

### Methods

#### Isolation of quinclorac-degrading endophytic bacteria

We collected the tobacco from a field with long-term usage of quinclorac. Then, the cleaned tobacco root was surface-sterilized sequentially using: 75% (v/v) ethanol for 2 min, ultrapure water three times with 1 min each and 1% mercuric chloride for 1 min. Finally the surface-sterilized tobacco root was washed by sterile ultrapure water three times again to clean sterilization agent residues. To confirm the success of surface sterilization, we inoculated the ultrapure water from the final rinse on petri dish with LB agar medium. The tobacco root was considered clean if no bacterial colony was found on petri dish after inoculation.

We grounded 0.3 g fresh root with 10 mL sterile ultrapure water using a sterile mortar. We spread the grinding fluid (50 ul) on a petri dish of mineral salt agar medium that contains 20 mg/L quinclorac. We cultured it in an incubator at 28°C for three days. Bacterial colonies with clear zones were selected as potential quinclorac-degrading bacteria.

#### Characterization of quinclorac-degrading strain Q3

The identification of strain Q3 was performed according to Bergey's Manual of Determinative Bacteriology. First, we studied the morphological characteristics of strain Q3. We streaked Q3 on solid LB media to observe its colonial morphology. We also cultured strain Q3 in liquid LB media for 28 h and scanned the strain morphology using a scanning electron microscope. Then, we assessed basic physiological and biochemical characteristics of strain Q3. We performed 71 carbon source utilization tests and 23 chemical-sensitivity tests of strain Q3 using a GNIII microplate (96 holes) from Biolog Corporation (USA).

#### 16S rDNA sequencing and phylogenetic analysis

DNA of strain Q3 was extracted and purified with a commercial DNA extraction kit (TransGen Biotech, China). Then, the 16S rDNA was amplified by polymerase chain reaction (PCR) using general bacterial primer 27F (5′-AGAGTTTGATCCTGGCTCAG-3′) and 1492R (5′-GGTTACCTTGTTACGACTT-3′). The PCR mixture contained 2.0 µL 10×Ex Taq buffer, 1.6 µL MgCl_2_(25 mM), 1.6 µL dNTP (2.5 mM), 1.0 µL of each primer, 0.5 µL DNA template, 0.2 µL Taq DNA polymerase (10000 U.mL^−1^), and 12.1 µL ddH_2_O in a total volume of 20 µL. The PCR amplification process consisted of one cycle of 5 min denaturation and Taq activation at 95°C, 40 cycles with each consisting of 30 s at 95°C, 30 s at 55°C, and 90 s at 72°C, and a final extension cycle of 10 min at 72°C. The PCR products were first verified by agarose gel electrophoresis. Then, purified PCR products were sequenced by Shanghai Majorbio Bio-pharm Technology Co., Ltd. The 16s rDNA sequence was aligned with published sequences in GenBank using BLAST program, and phylogenies was analyzed using MEGA (version 5.0) software.

#### Measurement of *Bacillus megaterium* Q3 Growth

*Bacillus megaterium* Q3 was cultivated in liquid LB culture for 24 h in advance. The culture liquid was then centrifuged at 2152×g for 3 min to collect bacteria cells. The collected cells were washed three times to remove remaining LB culture. Then, the cells were inoculated in a flask containing degrading culture for subsequent study.

Growth of Q3 was determined by the spectrophotometric method operated under 600 nm. First, 0.1 mL culture liquid was transferred into a centrifuge tube and centrifuged at 2152×g for 3 min. The cells were collected and washed with 0.3% sterile saline three times, and then fully resuspended in 1 mL 0.3% sterile normal saline. The OD_600_ value was measured by a spectrophotometer (Shimadzu, Japan).

#### Measurement of quinclorac degradation

The degradation of quinclorac was monitored by high performance liquid chromatography (HPLC) (Shimadzu, Japan) equipped with a SPD-20A UV detector and an Athena C_18_ column (CNW, 5 µm, 4.6 mm×250 mm) using a method developed by Wang et al. [17]. For each measurement, 1 mL degradation liquid was thoroughly mixed with 0.25 mL chloroform-n-butyl alcohol (volume ratio 4∶1) for 30 min and centrifuged at 12396×g for 5 min. Then, the supernatant was filtered through a 0.22 µm filter membrane and detected by HPLC. Water (containing 0.2% acetic acid)/methanol (40/60, v/v) mixture was used as effluent with a flow rate of 0.8 mL/min for HPLC. The detections were performed at a wavelength of 240 nm with column temperature at 30°C. The injection volume was 20 µL.

### Measurement of quinclorac metabolites

#### Extraction of degradation products

2 mL degradation liquid was transferred to a centrifuge tube and centrifuged at 12396×g for 5 min after deproteinization. We took the supernatant and added 4 mL ethyl acetate to extract the degradation products. Then, we added saturated sodium chloride to make the mixed liquid separate into two layers. The ethyl acetate layer was taken and dewatered by adding anhydrous sodium sulfate; then, the ethyl acetate layer was concentrated before examination by an ACQUITY/ZQ 4000 HPLC-MS/MS (Waters, America).

#### Detection condition of HPLC

ACQUITY UPLC BEH C_18_ column (50×2.1 mm,1.7 µm)was used. 10 mmol/L ammonium acetate (containing 0.1% formic acid)-methanol was used as effluent with a flow rate of 0.2 mL/min. The sample room temperature was 10°C and the column temperature was set to 30°C. The injection volume was 10 µL.

#### Detection condition of MS/MS

The mass spectrometer was operated in the positive polarity mode. The MS/MS interface was performed under the following conditions: gas temperature of 350°C, cone voltage of 25 V, collision energy of 20 eV, and a capillary voltage of 3000 V. Full scans were conducted from 0 min to 8.0 min with scan time of 0.1 second. The scan scope was 100 to 290 m/z.

#### Pot experiment

The pot cultivation of tobacco were conducted under three conditions: 1) soil with quinclorac and Q3 added (bioremediation treatment); 2) soil with quinclorac but no Q3 added (phytotoxicity treatment and marked as YHCK); 3) soil without quinclorac and no Q3 added (blank control and marked as CK). Three replicate experiments were conducted in each case.

We collected quinclorac-contaminated soil from the tobacco field of Hunan Agricultural University. Rice had been planted in the field previously and quinclorac was used to control weeds with dose of 40 g (active ingredient)/667 m^2^. The quinclorac concentration in collected soil was 0.04 mg/kg. After cultured in liquid LB medium for 24 h, 10 ml of Q3 fermentation broth was applied to soil in pot for seven days before tobacco was transplanted. Each pot had one tobacco seedling transplanted into its soil. The tobacco was cultured in a greenhouse after transplanting. Obvious phytotoxicity symptom was observed within approximately 30 days after transplanting. Then, the first investigation was conducted on leaf length, leaf width, and height of each tobacco plant. The second investigation was conducted about 15 days after the first one.

## Results

### Identification and Characterization of quinclorac degrading bacteria, *Bacillus megaterium* Q3

We cultured ground quinclorac-contaminated tobacco root for three days. We were able to isolate six bacteria strains that colonize with clear zones. Those six strains were denoted as strain Q1–Q6. We tested the quinclorac degradation capabilities of those six strains (data not shown). Among the six strains, strain Q3 had the highest quinclorac degradation rate compared to the other five strains. Thus, we focused our further research on strain Q3.

**Figure 1:**
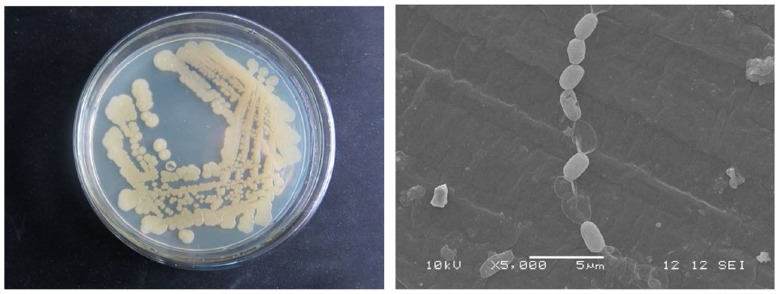
Morphological characteristics of strain Q3. Colony morphology on LB plate (left) and cell morphology under electron microscope (right).

We first studied the morphological characteristics of strain Q3. The colonial morphology of strain Q3 on LB plates was thin flat, faint yellow, opaque, round with smooth edge and were about 1.2∼3 mm in diameter (Figure 1 left). The cell morphology of strain Q3 under scanning electron microscope was short rod-like and 1.2∼1.5×1.9∼3.7 µm in size, which occurred singly or in short chains (Figure 1 right).

Then, we assessed the basic physiological and biochemical characteristics of strain Q3. Gram staining test showed that strain Q3 is a Gram-positive bacterium. Thus, we used Biolog GN III microplate (containing a 71 carbon source utilization test and 23 chemical-sensitivity test) to determine the Q3 strain's substrate utilization. After 18 h incubation, the Biolog GN action profile of Q3 was similar to that of *Bacillus megaterium* with a SIM index of 0.673, a PROB index of 0.977,and a DIST value of 5.774.

**Figure 2:**
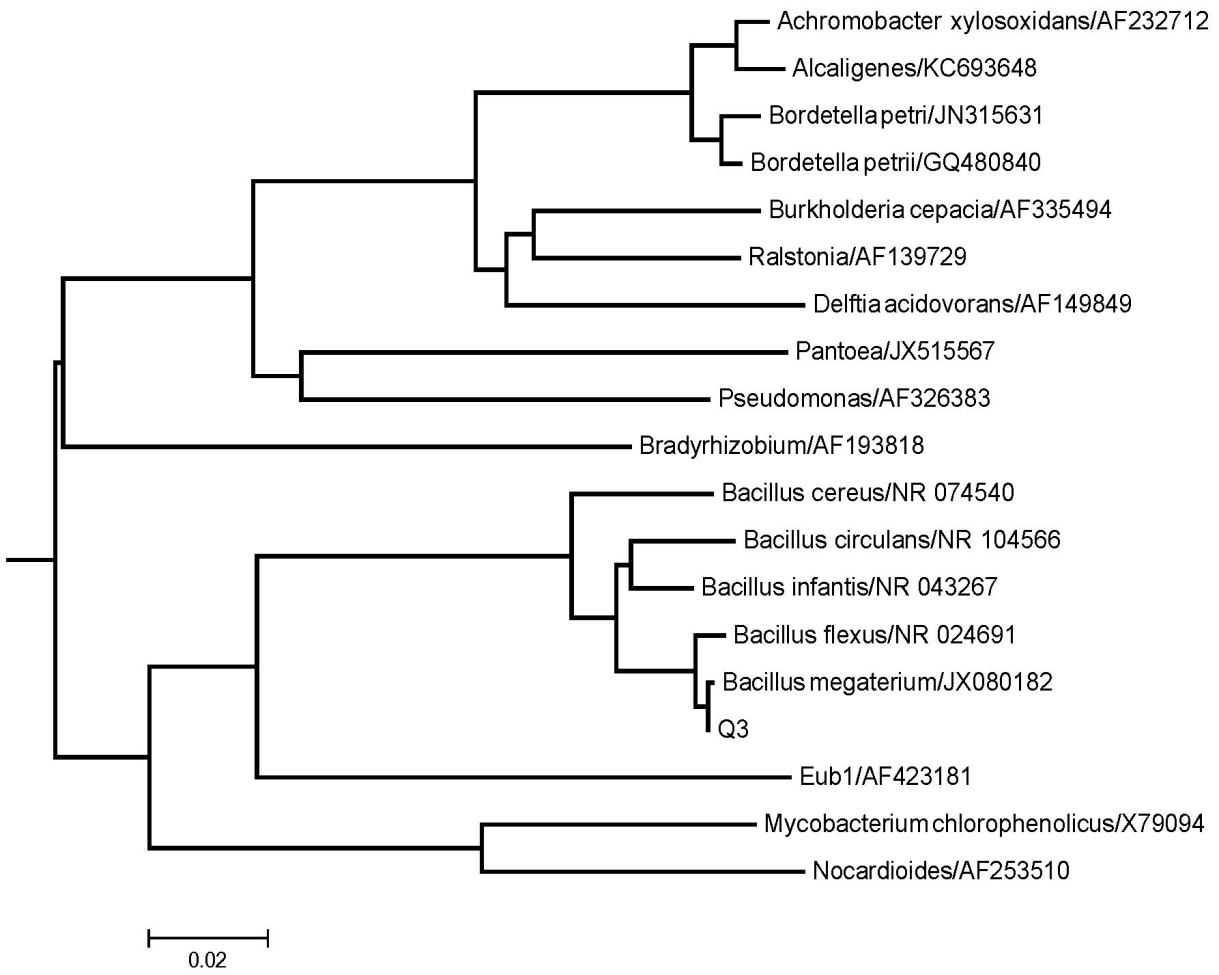
The phylogenetic tree based on 16S rDNA of strain Q3 and those of herbicide-degrading species. Q3 and other two quinclorac degrading bacteria, such as *Burkholderia cepacia* and *Bordetella petrii* are in different branches of the tree.

To further classify Q3, we sequenced part of its 16S rDNA sequence. We obtained a 1287 bp amplification fragment of 16s rDNA of strain Q3 by PCR. We compared this partial 16s rDNA sequence with sequences in the Genbank Database. The 16S rDNA sequence of Q3 was identical to that of *Bacillus megaterium* N1564-A29 (JX080182). The result was consistent with morphological characteristics, physiological and biochemical characteristics. We then aligned the 16 s rDNA sequences with those of several herbicide degrading bacteria and constructed a phylogenetic tree (Figure 2). The phylogenetic tree clearly showed that Q3 belongs to the same branch of *Bacillus megaterium* N1564-A29 and was obviously different from other quinclorac degrading bacteria, such as *Burkholderia cepacia* [10] and *Bordetella petrii* [13]. Therefore, we identified and designated isolated strain Q3 as *Bacillus megaterium*.

### Identification of optimal degradation conditions of Q3

#### Effect of temperature on degradation of quinclorac

**Figure 3:**
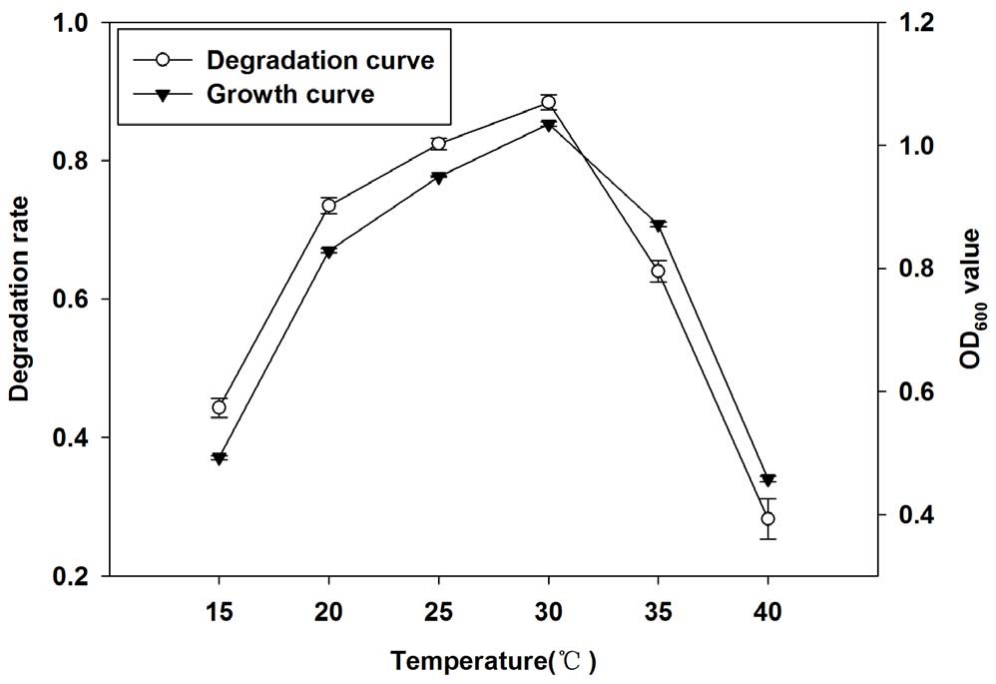
Effect of temperature on degradation of quinclorac and growth of Q3. Both growth and quinclorac degradation of Q3 reached peak values at temperature of 30°C.

We incubated the Q3 with degradation culture media at 15°C, 20°C, 25°C, 30°C, 35°C and 40°C separately. We measured the degradation rate of quinclorac and growth of Q3 after 7 days of incubation. Figure 3 plots the growth rate of Q3 and the degradation rate of quinclorac with temperatures. It showed that temperature had a significant effect on both Q3 growth and degradation of quinclorac. When the culture temperature increased from 15°C to 30°C, growth of Q3 and degradation of quinclorac also increased. At the temperature of 30°C, both OD_600_ value and quinclorac degradation rate of Q3 reached peak values, which were 1.03 and 88.4%, respectively. When the temperature was increased above 30°C, both growth of Q3 and degradation of quinclorac decreased as temperature increased. So, the optimal temperature for the growth and quinclorac degradation of Q3 was 30°C.

#### Effect of pH on degradation of quinclorac

**Figure 4:**
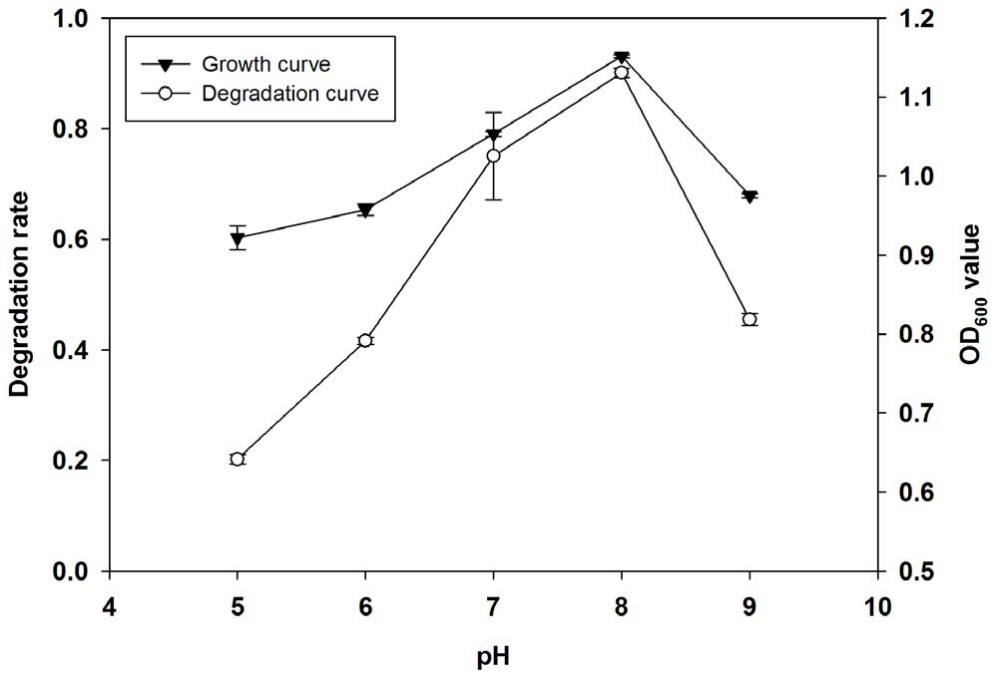
Effect of pH on degradation of quinclorac and growth of Q3. Both growth and quinclorac degradation of Q3 reached peak values at pH of 8.

We set the pH of degradation culture medium to 5, 6, 7, 8 and 9, respectively, and incubated the Q3 at 30°C in incubator for seven days. Then, we measured the degradation rate of quinclorac and growth of Q3. Figure 4 plots the growth rate of Q3 and degradation rate of quinclorac with pH. It shows when the pH of culture increased from 5 to 8 as both growth of Q3 and degradation of quinclorac also increased. Q3 reached the highest growth rate and highest quinclorac degradation rate at pH 8 when the OD_600_ value was 1.13 and quinclorac degradation rate was 93.1%, respectively. Growth of strain Q3 and degradation of quinclorac sharply decreased when culture medium was set to both low and high pH levels. So, the optimal pH for growth and quinclorac degradation of Q3 was 8.0.

#### Effect of inoculation size on degradation of quinclorac

**Figure 5:**
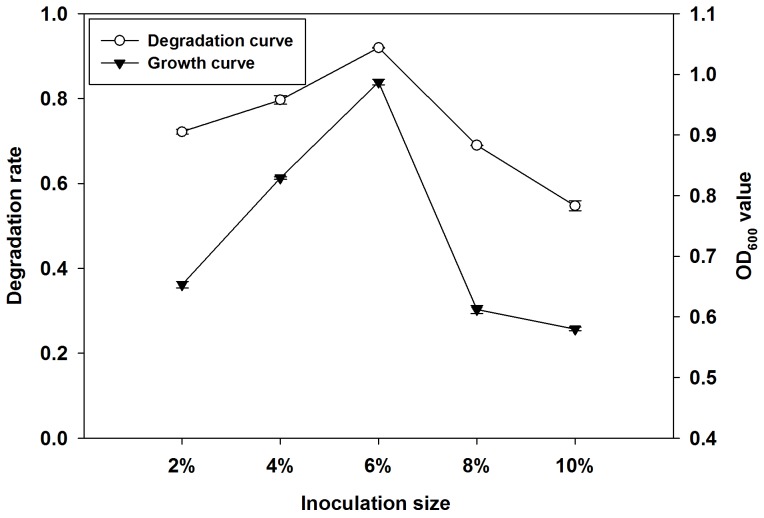
Effect of inoculation size on degradation of quinclorac and growth of Q3. Both growth and quinclorac degradation of Q3 reached peak values when inoculation size was 6%.

To study the effect of inoculation size on growth and quinclorac degradation of Q3, we inoculated 2%, 4%, 6%, 8% and 10% (volume ratio) of Q3 into degradation medium, respectively. Then, we measured the quinclorac degradation rate and growth of Q3 after 7 days of incubation. Figure 5 shows the effect of inoculation size on Q3 growth and degradation of quinclorac. Figure 5 also illustrates the growth increase of both Q3 and the quinclorac degradation rate when inoculation size increased from 2% to 6%. The OD_600_ value was.0.99 and quinclorac degradation rate was 91.9% at inoculation size of 6%, respectively. However, both growth and quinclorac degradation of Q3 decreased when the inoculation size was increased above 6%. So the optimal inoculation size for Q3 was 6%.

#### Effect of initial quinclorac concentration on degradation of quinclorac

**Figure 6:**
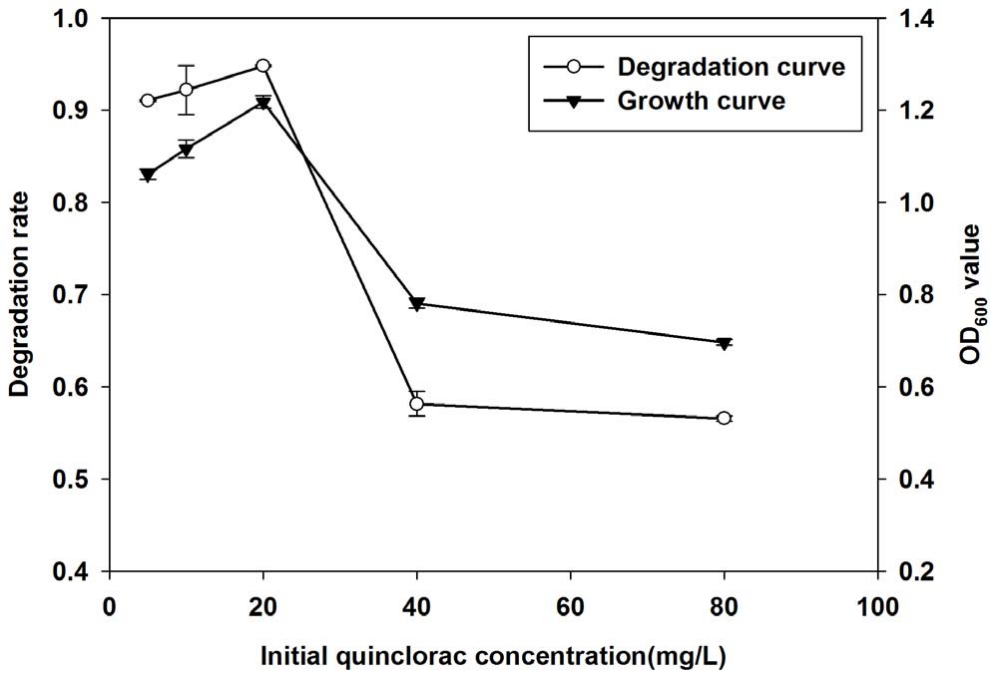
Effect of initial quinclorac concentration on Q3 growth and degradation of quinclorac. Both growth and quinclorac degradation of Q3 reached peak values when initial quinclorac concentration was 20 mg/L.

To study the effect of the initial quinclorac concentration on degradation and Q3 growth, we incubated Q3 on different degradation mediums with initial quinclorac concentrations of 5, 10, 20, 40 and 80 mg/L. Then, we measured the degradation rate of quinclorac and growth rate of Q3 after 7 days. Figure 6 shows that Q3 can grow well when initial quinclorac concentration is less than 40 mg/L. However, Q3 cannot grow well when the initial quinclorac concentration reaches 40 mg/L. Meanwhile, the degradation rate of quinclorac correlated with the growth of Q3. Q3 had a high degradation rate when the initial quinclorac concentration was less than 40 mg/L. The degradation rate reached a peak value of 94.8% when initial quinclorac concentration was 20 mg/L. The degradation rate reduced dramatically when the initial quinclorac concentration was beyond 40 mg/L.

#### Effect of degradation time on quinclorac degradation

**Figure 7:**
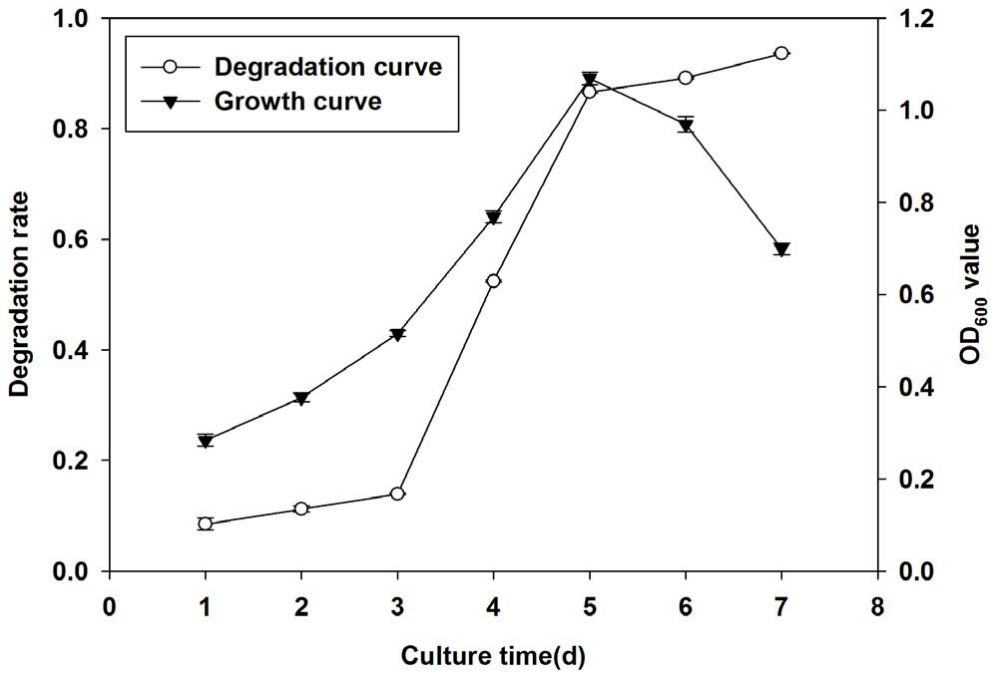
The growth and quinclorac degradation curve of Q3 at optimal condition. The degradation of quinclorac is highly correlated with the growth of Q3.

We incubated Q3 under optimal conditions setting the temperature at 30°C, pH at 8, inoculation size at 6%, and initial quniclorac concentration at 20 mg/L. We measured the degradation rate of quinclorac and growth of Q3 for seven days. Figure 7 shows how the degradation rate of quinclorac increased slowly during the first three days. Then, the degradation rate increased sharply on the fourth and fifth day. After that, the degradation rate only slightly increased. The degradation rate on the seventh day was 93.6%. Meanwhile, OD_600_ value of Q3 showed a gradual increase and reached its peak value by the fifth day whereupon the growth rate of Q3 began to decline.

### Analysis of quinclorac degradation products of Q3

**Figure 8:**
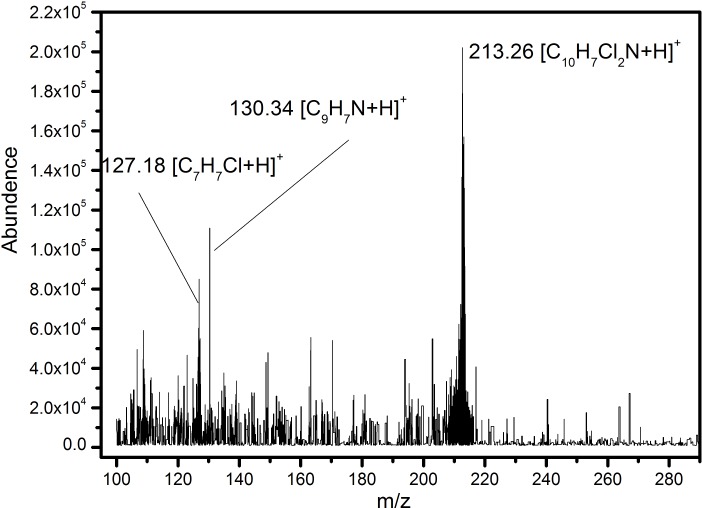
Mass spectrum of quinclorac degradation products of Q3. The molecular formula of quinclorac and three degradation products are labeled.

We isolated the degradation products of quinclorac and analyzed those using LC-MS/MS. Three major degradation products were detected after Q3 was incubated in degradation medium for three days. Figure 8 shows the mass spectrum of degradation products on Day 3. The peak at 243.13 *m/z* is the original quinclorac. The peak at 213.29 *m/z* is resolved as 3, 7-dichloro-8-methyl-quinoline. The peak at 208.09 *m/z* is resolved as 3-chlorin-8-quinoline-carboxylic. And the peak at 191.10 *m/z* is resolved as 8-quinoline-carboxylic. The three degradation products were created separately with a retention time of about 2.00 min.

### Bioremediation of quinclorac phytotoxicity by Q3

**Figure 9:**
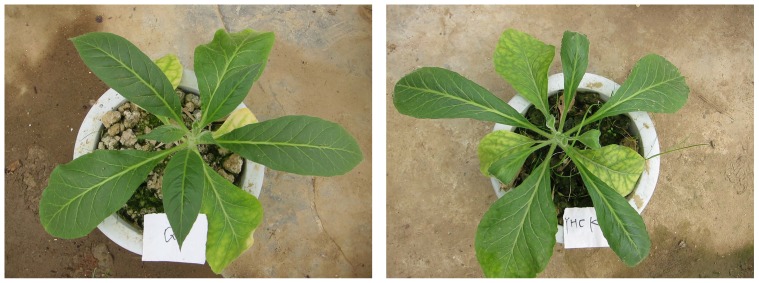
Bioremediation of strain Q3 (left) and phytotoxicity of quinclorac (right) on tobacco. The tobacco grew much better in Q3 added soil (left) than in soil without Q3, while soil in both containers were contaminated by quinclorac.

To examine the potential usage of Q3 as a bioremediation for quinclorac phytotoxicity, we compared the growth of tobacco in pots with different kinds of soil: soil with quinclorac and Q3 added, soil with quinclorac but no Q3 added, and soil without quinclorac and no Q3 added as control. Table 1 compared the leaf length, leaf width, and plant height of tobacco in soils with and without Q3 bioremediation. The results showed that Q3 can significantly reduce tobacco phytotoxicity. After having been transplanted for 30 days in the soil with quinclorac (0.04 mg/L) but no Q3, the leaf length, leaf width and plant height of tobacco were all significantly inhibited, which were only 83.18%, 64.75% and 67.67% of the growth rate for those in the control group. In contrast, in soil with quinclorac and Q3, leaf length, leaf width and plant height of tobacco recovered to 89.49%, 92.14% and 93.64% of the growth rate for those plants in control group. After having been transplanted for 45 days, the recovery rate of leaf length, leaf width and plant height of tobacco in Q3 bioremediated quinclorac soil was 93.40%, 91.70% and 92.29, respectively. While without Q3 bioremediation, the leaf length, leaf width and plant height of tobacco were further affected by quinclorac and decreased to 79.25%, 40.09% and 74.51% of growth rates in control group. The results also showed that inhibition of quinclorac on leaf width of tobacco was more serious than inhibition on leaf length and plant height (Figure 9).

**Table 1:** Bioremediation of strain Q3 on leaf length, leaf width and plant height of tobacco.

	quinclorac+Q3/30days (mean±sd)	quinclorac/30days (mean±sd)	quinclorac+Q3/45days (mean±sd)	quinclorac/45days (mean±sd)
Leaf length	89.49±2.71	83.18±0.68	93.40±0.64	79.25±0.78
Leaf width	92.14±3.24	64.75±2.46	91.70±1.71	40.09±0.59
Plant height	93.64±4.95	67.67±4.20	92.29±4.55	74.51±2.86

Note: Values are the means and standard deviation (sd) of three replicates. Values are percentage of leaf length, leaf width and plant height of treatments compared with controls without quinclorac phytotoxicity.

## Discussion

In this study, we isolated and identified an endophytic quinclorac-degrading bacterium strain Q3 that can effectively degrade quinclorac. All previously quinclorac degrading bacteria were isolated from soil. The initial degrading concentrations of those bacteria are much higher than the quinclorac concentration in rice fields [16]. On the other hand, the Q3 strain is capable of degrading quinclorac in low concentration. Furthermore, our indoor pot experiments showed that Q3 can be a good bioremediation for tobacco quinclorac phytotoxicity. However, it is unclear if the strain Q3 still had the same degradation and bioremediation efficiency when it is used in natural environment. We will investigate the intervention of Q3 in actual tobacco and rice fields in detail in the future.

Similar to previously identified four quinclorac degradation strains, Q3 reached peak degradation rate at temperature 30°C. However, the four strains identified before prefer the neutral condition at pH of 7, and the Q3 prefers the alkaline condition at pH of 8 for quinclorac degradation.

Endophytic strains usually not only exist in plants, but also conduct a stable breeding in soil, so endophytic strains could degrade pesticides in both soil and plants [18], [19]. Meanwhile, endophytic strains could produce indole acetic acid (IAA) and siderophores to promote plant establishment and enhance plant growth and development, thus promoting phytoremediation of pesticide [18]. Therefore, endophytic degrading bacterium may be more efficient in pesticide degradation [18], [19].

**Figure 10:**
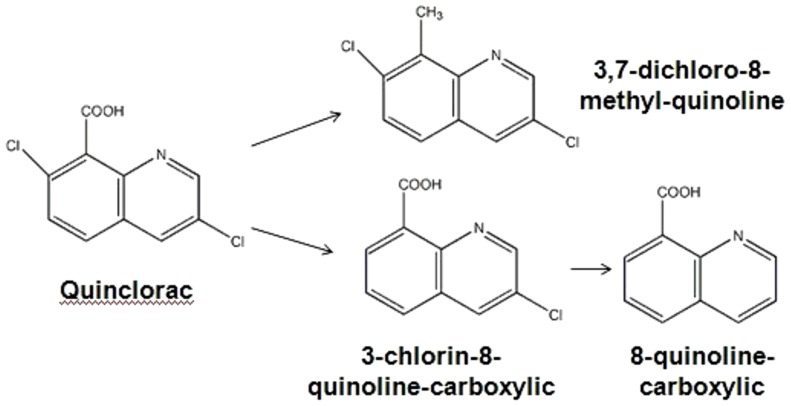
Possible quinclorac degradation pathways of Q3. The pathways were proposed based on the resolved quinclorac degradation products from mass spectrum.

The major degradation products by Q3 were identified as 3, 7-dichloro-8-methyl-quinoline, 3-chlorin-8-quinoline-carboxylic and 8-quinoline-carboxylic. This is different from the results of previous study [10] where Lü et al. once reported that *Burkholderia cepacia* strain WZ1 could degrade quinclorac to 3,7-dichloro-8-quinoline and 2-choro,1,4- benzenedicarboxylic acid, respectively. Based on the results of mass spectrum, we hypothesized that the Q3 may employ multiple pathways to degrade quinclorac. One possible pathway is through the reduction of carboxyl, and the other pathway is through dechlorination of quinclorac (Figure 10). The degradation pathway of Q3 was different from previous reported by Lü et al. [10]. Due to lack of standard substance and failure to isolate a pure degradation product, we were not able to make qualitative and quantitative analysis of the degradation products. Furthermore, how strain Q3 degrades quinclorac and what the key enzyme is in the process of quinclorac degradation remains unknown. In our future work, we will sequence the whole genome of strain Q3 and elucidate the key enzymes for quinclorac degradation and transcription factors that control the quinclorac degradation.
